# The Automobile in Country Practice

**Published:** 1900-09-01

**Authors:** 


### THE AUTOMOBILE IN COUNTRY PRACTICE.

Coming fresh from Paris, where the automobile seems to reign supreme, it is rather with a shock that one reads in the New York *Medical News* an article by Dr. A. D. Hard, a practitioner who evidently has had much practical experience on the subject, that whatever may be the future of automobilism, the country doctor cannot as yet successfully use any automobile now on the market to make his regular trips over average roads at all times of the year, and that an investment made upon different representations will result in failure and disappointment. No doubt the roads have much to do with the problem, and the roads in Dakota may not be very good. The "chauffeur" who glides easily about the splendid main roads by which France is intersected in every direction has things all his own way, and no one can observe the ease, the celerity, and the apparent safety with which automobiles of all description—bicycles, voiturettes, and ponderous vehicles of many kinds—traverse the streets of Paris and the roads of the country for miles around, without feeling sure that the doctor, with his constant demand for rapid locomotion, ought in many instances to be able to get out of the automobile just the thing he wants. But the practical man from Dakota says no. He holds that in the first place the roads stand in the way, next that the mechanism is too uncertain, and lastly, that in very severe weather the automobile becomes inoperative, in the case of petrol from lack of vapour, and in case of steam from frozen pipes; while electricity, the only other available source of power, is out of the question for practical purposes, in consequence of the necessity of recharging the accumulators at frequent intervals. All this is very depressing to those who had pinned their hopes upon automobilism as one way out of the ever-pressing problem of the stable account. But we are not yet convinced. Everyone will admit that there are roads on which a motor could not safely be taken—indeed, could not be taken at all; and that now and again there is weather in which starting a motor might be a difficulty, although a modern motor once started ought to go even in very cold weather. But it must be remembered that there are roads on which one cannot take a carriage with any comfort, and that there are days when one's horses, if they are worth anything, don't go out. Practically, it seems to become a question chiefly of roads. For the elderly or delicate man, who must always drive up to the door, the ordinary means of locomotion will probably always remain the best; but for the strong man, who is ready to use his legs when occasion requires, the automobile, as it seems to us, offers great advantages in districts where the roads are decent and the gradients are not too heavy, allowing him with great facility to get within easy walking distance of most of the places he wants to visit.

